# Current Status of Telerehabilitation Services in Low-Middle Income Countries - A Scoping Review

**DOI:** 10.63144/ijt.2025.6724

**Published:** 2025-12-12

**Authors:** Rehana Parvin, John Parsons, Karen Day

**Affiliations:** 1 School of Population Health, University of Auckland, New Zealand; 2 School of Nursing, University of Auckland, New Zealand

**Keywords:** Developing countries, Disabled people, Low-middle income countries (LMICs), Telehealth, Telerehabilitation

## Abstract

Telerehabilitation adoption in low-middle income countries (LMICs) accelerated during COVID-19 pandemic, promoting a surge in telehealth initiatives, many of which remain unexplored. This scoping review aims to assess the current state of telerehabilitation services in LMICs across Asia and Africa using Arksey and O’Malley’s five-stage framework with PRISMA-ScR guidelines. Two reviews were conducted: one covering from 2012 to 2022 and another from 2023 to January 2025, yielding a total of 87 relevant articles. The key themes that emerged from the collected literature include current telerehabilitation status, pandemic utilization, implementation challenges, user understanding of telerehabilitation along with their perceptions and practices, and feasibility together with legal-ethical aspects and acceptability. Despite limited ICT skills and infrastructure challenges, users reported positive experiences, primarily using mobile phones and video conferences. However, privacy concerns along with digital literacy issues remain. Although many nations adopted WHO guidelines and donor support, formal policies and sustainable implementation strategies are still lacking. The findings underscore the need for context specific and sustainable frameworks to strengthen telerehabilitation in LMICs.

Telehealth services now receive global recognition as an essential healthcare practice (Macabasag et al., 2016). The worldwide rapid spread of COVID-19 made telehealth services transition from optional to mainstream healthcare delivery (Leochico, 2020; Yasmin et al., 2020). Telerehabilitation exists within telehealth boundaries as a rehabilitation and habilitation system which provides assessment and monitoring, therapeutic intervention, education, consultation, and counseling for people with chronic conditions and/or disabilities (Jones et al., 2020; Nizeyimana et al., 2022). Nizeyimana et al. (2022) performed a scoping review to analyze telerehabilitation feasibility alongside its cost-effectiveness, accessibility, and impact. In their study the authors used the term telerehabilitation to include both telehealth and telemedicine functionalities. The current scoping review adopted the same strategy as the search results were insufficient for the term “telerehabilitation” alone.

Healthcare systems of high-income countries (HICs) have started implementing telehealth to improve service provision in remote areas. Multiple low- and middle-income countries (LMICs) adopted similar healthcare strategies after observing positive outcomes in bridging healthcare inequities and improving health and quality of life (Macabasag et al., 2016; Mahmoud et al., 2022). The COVID-19 crisis led to a dramatic surge in telerehabilitation usage. The services of India along with Lebanon and China expanded their operations quickly (Mahmoud et al., 2022). According to Ong et al. (2022) telerehabilitation services witnessed a 44% increase in usage over five years. Furthermore, mobile health applications (mhealth) expanded significantly throughout Southeast Asia during the past decade (Sabrina & Defi, 2021).

Recent studies further demonstrate the effectiveness of telerehabilitation in LMICs. For instance, Estela-Zape et al. (2025) reported that post-COVID-19 patients experienced better respiratory and functional results through telerehabilitation services while Munce (2025) highlighted emerging tools like virtual reality (VR) and artificial intelligence (AI) to enhance tailored rehabilitation delivery. Despite these advantages, telerehabilitation adoption was slow before the pandemic and remained limited in numerous LMICs due to lack of infrastructure and training (Odetunde et al., 2024; Sharma et al., 2024). Nonetheless, multiple studies demonstrate that telerehabilitation offers a long-term framework for future healthcare systems rather than being a temporary response to emergencies (Anil et al., 2023).

Evidence indicates this approach was successful in the SARS (2003), MERS (2015), and Ebola (2014 – 2016) outbreaks (Mahmoud et al., 2022). However, many of the telehealth initiatives in LMICs failed to sustain because of expense, insufficient infrastructure, and uncoordinated implementation plans (Macabasag et al., 2016; Rahman, 2025).

This scoping review aims to map the existing available information about telerehabilitation practices in LMICs. The research aims to determine the obstacles that healthcare professionals face when using telerehabilitation while also analyzing its acceptance levels.

## Methodology

The research design uses the scoping review approach (Arksey & O’Malley, 2005) to explore the current state of telerehabilitation in LMICs. According to Nizeyimana et al. (2022), research studies conducted through scoping reviews identify and document existing knowledge without performing quality assessment.

The research employs the refined Joanna Briggs Institute framework (Peters et al., 2017) while following Arksey and O’Malley’s approach (Arksey & O’Malley, 2005). The research questions stem from Population, Concept and Context (PCC) elements (Peters et al., 2017). The population includes individuals of all ages and physical conditions from LMIC residents while including studies about healthcare providers and telehealth related documents. The concept encompasses multiple telehealth elements that include infrastructure barriers, opportunities, perceptions and other relevant topics. The contextual relevance to LMICs or developing countries is a criterion for this study as well.

The method involved the following steps.

1. Formulating Research Questions: This was based on the PCC framework to develop keywords for database searches.
2. Identifying Relevant Studies: The researchers performed database searches across PubMed, Scopus, and EBSCOhost platforms which contain CINAHL, Academic Search Premier, MEDLINE, Google Scholar, Embase, Cochrane, Web of Science, IEEEXplore, LILACS, and AIM using the Boolean operators “AND” and “OR” (Appendix). The initial search included English language studies from 2012 to 2022 with the addition of supplementary searches from 2023 to early 2025.
3. Study Selection: All relevant references were collected from electronic databases and managed using EndNote^TM^ version 20. The elimination process began with removing duplicate records before screening title and abstract information for inclusion criteria. The researchers retrieved all eligible full-text studies for individual assessment. The compiled list of potential articles followed the Preferred Reporting Items for Systematic Reviews and Meta-Analyses for Scoping Reviews (PRISMA-ScR) format (PRISMA, 2018). This study did not perform quality appraisal during the evaluation process. The PRISMA – ScR diagram used in systematic reviews was modified to display the scoping review process which helped select the final papers for analysis.
4. Data Charting: An Excel spreadsheet data charting form was designed to answer the research questions. Researchers organized the extracted data by using categories such as authors’ names, publication years, study type, study location, research objectives and study design, barriers, opportunities, total telehealth project, duration and study conclusions.
5. Summarizing Data: The gathered information was grouped under categories/themes that included telehealth communication methods (e.g., video conferencing, mobile, store forward) purposes, financial aspects and obstacles, acceptance and feasibility.

## Results

**Figure 1:**
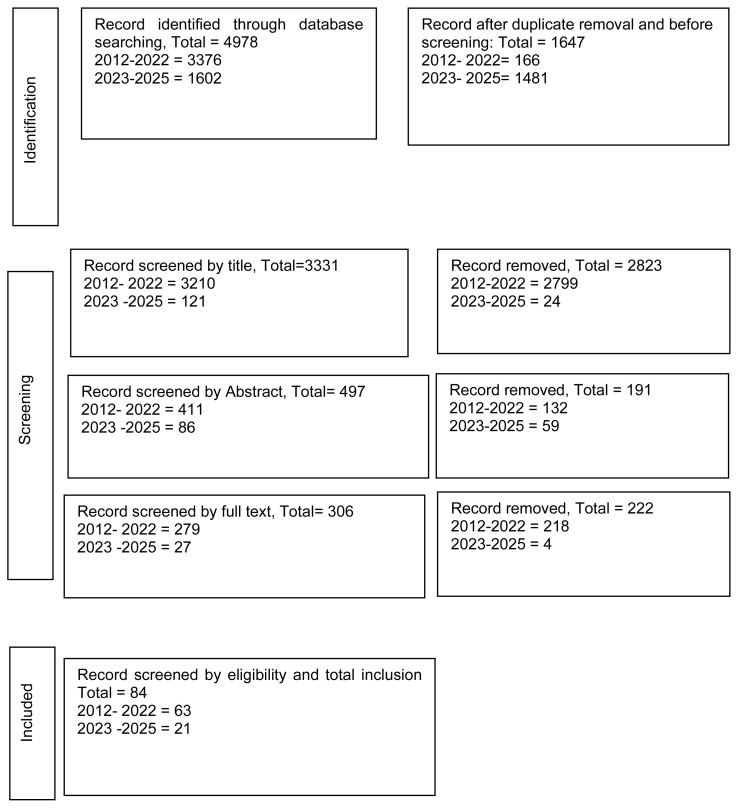
PRISMA Flowchart

The scoping review began with 2,062 articles discovered through the search protocol. After eliminating 166 duplicate articles, the screening process identified 1,907 articles which led to the selection of 390 abstracts for evaluation resulting in 266 full-text articles. The assessment of full-text articles based on inclusion criteria resulted in the selection of 70 studies for the first phase of the scoping review. The updated search for 2023 to 2025 added 1084 articles to the pool. From this updated search, 102 articles were selected through title screening and 78 articles from abstract review. The full-text evaluation process added 17 more studies that met the inclusion criteria to the final analysis, resulting in a total of 87 articles for this scoping review. Following journal feedback, an additional search across other relevant databases identified 14 more studies and excluded 17 studies from high-income countries. The final analysis, therefore, included 84 studies (Figure 1).

**Figure 2:**
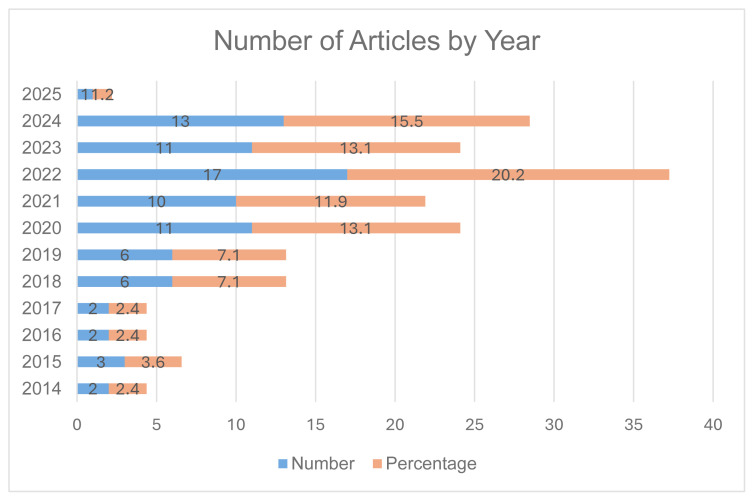
Distribution of Articles by Year

**Figure 3:**
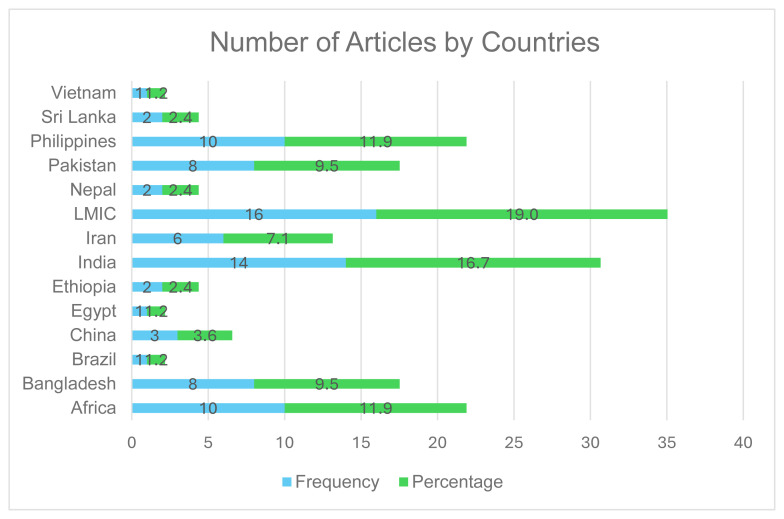
Distribution of Articles by Countries

The highest number of articles was published in recent years, with 2022 representing the peak year with 20.5% (17) (Figure 2). Among the studies, 19.3% (16) focused on LMICs as their geographic area of interest. The top four countries for research publications were India, the Philippines, Pakistan, and Bangladesh (Figure 3). The studies from African nations (Ghana, Senegal, Zambia, Cameroon, and Tanzania) were combined into the “Africa” group.

Figure 2 illustrates the distribution of articles by publication year. According to this figure, the highest (20.5%, 17) was published in 2022. In contrast, only one article was retrieved from 2025 (1.2%) and three from 2015 (3.6%). From 2020 to 2023, a similar number of articles (13.1%, 11; 11.9%, 10) were retrieved. Additionally, a small number of articles (2, 2.4%) were published in the years 2014, 2016, and 2017.

Figure 3 highlights the number of articles by countries. The figure shows that the highest number of articles (16, 19.0%) are retrieved from LMICs in general. Among individual countries, Vietnam and Egypt (1,1.2%), Sri Lanka and Ethiopia (2,2.4%) and China (3, 3.6%) had the lowest number. India had the highest number of publications among the Asian countries, with 14 articles (16.7%), while 10 (12%) originated from African regions and Philippines. Bangladesh and Pakistan contributed 8 (9.5 %) articles each.

**Figure 4:**
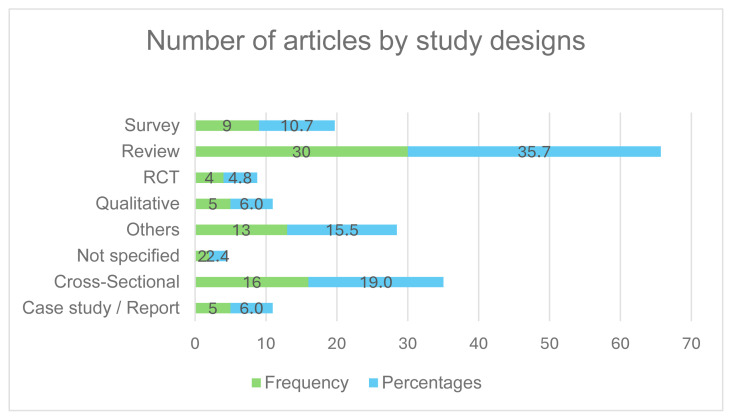
Distribution of Articles by Study Designs

Figure 4 shows that, among the selected articles, review articles comprised the largest percentage of studies at 35.7% (30), while cross-sectional studies followed at 19% (16), surveys at 10.9% (9) and qualitative studies and case study/report at 6% (5) each. Various other study designs, including randomised controlled trials, retrospective, prospective, pilot, and empirical studies, were also included in the research. The review articles addressed a wide range of topics and consisted of systematic (13), scoping (6), and narrative (3) reviews.

**Figure 5:**
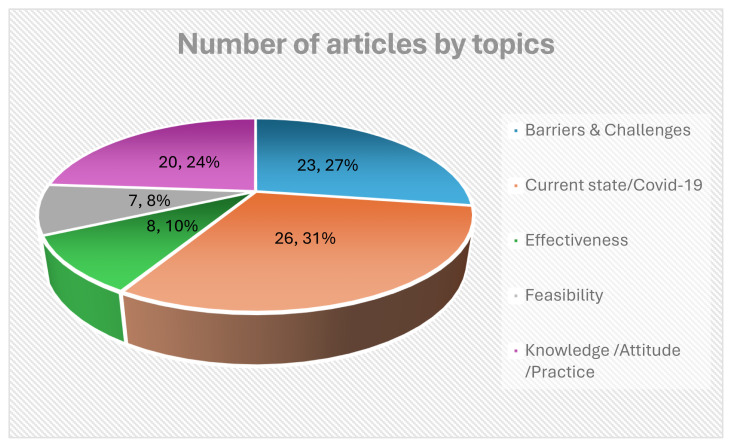
Distribution of Articles by Topics

Figure 5 illustrates the distribution of articles by topic covered. According to this pie chart, the highest 31% (26) focused on the current state of telerehabilitation. This was followed by 27% (23) addressing the barriers and challenges, and 24% (20) exploring knowledge, attitude and practice. The remaining 10% (8) and 8% (7) covered other topics like effectiveness and feasibility.

The review organized its key findings into five main categories that included country-specific situations and service availability, impact of the COVID-19 pandemic, reported barriers and challenges, knowledge, attitudes, and practices related to telerehabilitation, and recommendations for future policy and practice.

### Country-specific Current Status and Service Availability

Fifteen studies provided detailed, country-specific descriptions of their current status of telerehabilitation services. Of these, three are in Bangladesh, and two are in Pakistan and Ghana. The remaining studies cover countries such as India, Tanzania, Senegal, Vietnam, Sri Lanka, Philippines, Egypt, Cameroon and general LMICs. A summary of the country-specific findings is presented in Table 2.

### Current State and Policy Initiatives

Telerehabilitation has emerged as a critical component of health system strengthening in LMICs, particularly in response to the healthcare access gaps highlighted by the COVID-19 pandemic. The adoption and implementation of telerehabilitation, alongside policy support, varies greatly across different regions because of different levels of infrastructure and digital literacy as well as political commitment and socio-cultural readiness.

#### South Asia

In South Asia, India stands out for its structured implementation of telerehabilitation, especially during the COVID-19 pandemic through eSanjeevani, a cloud-based telemedicine platform that delivered remote medical consultations nationwide (Dash et al, 2021; Rajkumar et al., 2023).

The platform operates as a fundamental component of digital health service delivery through its connection to government hospitals and medical colleges. Beyond government effort, e-private centres surpassed 430 in number to offer telehealth services despite persistent digital literacy and connectivity issues (Chandwani & Dwivedi, 2015; Moorthy, 2021). Bangladesh demonstrated a multi-tiered approach to digital health, offering telehealth services through government services, SMS based maternity health services, and mobile-based health consultancy (Hoque, 2016). The outreach efforts of non-government organisations (NGOs) and mobile providers used Aponjon along with Manoshi and Healthline platforms (Alam et al., 2021), though policy coordination remains fragmented.

Telehealth development in Pakistan has not advanced past its initial stages. While telerehabilitation services are operated by mobile applications and video conferencing platforms, they lack both national-level guidelines and a policy framework for this field (Mahdi et al., 2021; Qureshi et al., 2014). Malik et al. (2024) emphasize the necessity of developing mHealth solutions that match Pakistan’s diverse cultural and linguistic characteristics and its limited digital resources. The authors support the establishment of partnerships between different stakeholders to maintain sustainable adoption. Nepal, in contrast, has been implementing telehealth services for some time. Its National Health Policy of 1990 established the basis for digital inclusion initiatives. The SWAp (Sector wide approach project) framework of this country (2015–2020) focused on providing healthcare services to rural women and children through its efforts to achieve both equity and access (Lamichhane, 2020)

At the regional level, Choudhury et al. (2024) suggest creating a cross-border telemedicine framework that utilizes India’s and China’s technological capabilities at the regional level. Their study identifies Sri Lanka’s lack of infrastructure and Pakistan’s inadequate healthcare workforce as obstacles to both regional connectivity and standardized service delivery.

#### Africa

African nations have embraced this service with various levels of maturity. For instance, 22 eHealth projects in Ghana are currently in the pilot phase which are largely mobile driven, and donor supported. Vodafone’s Healthline initiative functions as a primary communication channel to fill healthcare awareness gaps throughout the country (Afarikumah, 2014). Rural areas continue to face both limited network connectivity and insufficient medical staff (Mensah et al., 2023).

Cameroon’s eHealth system demonstrates rapid growth in its rural service delivery network. Kobi et al. (2024) demonstrates an emerging but expanding telemedicine environment across the country supported by government and private enthusiasm. The accessibility gap between urban and rural areas regarding technology and healthcare delivery, however, remains substantial. The growing physician adoption of telemedicine tools described by Kobi et al. (2024), as reported by Bawak and Kamdjong (2018), requires specific rural-focused approaches to achieve equitable service expansion.

In Nigeria, telemedicine was introduced in 2007 through a project by the National Space Research and Development Agency and the Federal Ministry of Health. Despite some early adopters, widespread use remains low, though the COVID-19 pandemic has increased its necessity and encouraged gradual implementation (Ezeani et al., 2022). The Digital Health Strategy of Senegal for 2018–2023 serves as a formal national policy to transform healthcare through information and communications technology (ICT) integration, although its implementation remains slow, according to Sy (2018). Tanzania, by comparison, maintains a robust policy framework. The framework consists of three main elements, including the National eHealth Strategy from 2013–2018, HSSP (health sector strategic plan) III and IV and the Digital Health Investment Roadmap from 2017–2023, which supports more than 128 eHealth applications (Hamad, 2019).

#### Others

The healthcare sector in Iran has adopted digital health technologies, including AI, along with mHealth and telemedicine systems, across mental health and cancer treatment areas. The services mentioned by Shojaee-Mend et al. (2024) play an essential role in medical diagnosis as well as treatment and educational programs for physicians. Vietnam invested substantial financial resources during the pandemic to develop two mHealth applications, named *Bluezone* and *NCOVI*. However, Nguyen et al. (2022) report that these tools underperformed due to user interface and adoption challenges, underscoring the need for user-centric design.

#### Impact of the COVID-19 Pandemic

In this review, out of 84 articles, nine focused on COVID-19-related telerehabilitation. Four were published in 2021 alongside two articles from 2020, 2022–2023 and 2024 and only one study from 2021. Telehealth research studies investigated the current state of telehealth services (Dash et al., 2021; Kulatunga et al., 2020; Mansori et al., 2023; Rajkumar et al., 2023), while others investigated psycho-oncology (Estapé et al., 2022), neurorehabilitation (Srivastava et al., 2021) and a scoping literature review. Additional research analyzed the awareness levels (Nguyen et al., 2022) as well as adaptation and perception studies (Alsahil et al., 2024; Garg, et al., 2020a).

India, together with Saudi Arabia and Sri Lanka, started using telehealth services as the World Health Organisation (WHO) advised essential service strengthening for their health systems during the COVID-19 pandemic (Garg et al., 2020b).

A research survey in India demonstrated that tele-neurorehabilitation maintained both feasibility and acceptance during the COVID-19 pandemic (Surya & Someshwar, 2025). The Indian “eSanjeevani” telemedicine platform implemented telehealth programs with AI integration (Dash et al., 2021). Similarly, Sri Lanka concentrated on digital healthcare empowerment for its citizens (Kulatunga et al., 2020) during that period.

#### Reported Barriers and Challenges

This review outlines the obstacles that halt telerehabilitation projects from being implemented and sustained in LMICs, and the results are categorized under technical, organizational, human (staff/users), and legal and ethical categories (Table 3). The research studies employed semi-structured questionnaires (Gregory & Temboo, 2017; Han et al., 2019; Reyes et al., 2021) and literature reviews (Leochico et al., 2020; Kruse et al., 2019) to assess their outcomes. This review reveals that the barriers to implementation of telerehabilitation programs in LMICs are numerous and vary across infrastructure, policy, human resources, and ethical considerations (Table 3).

### Reported Knowledge, Attitude and Practice

#### Knowledge of Telerehabilitation

The research examined 20 studies to determine the understanding of telerehabilitation practices and attitudes along with implementation strategies. The knowledge level regarding telerehabilitation among healthcare staff in LMICs reveals wide variability. Multiple research studies found that healthcare practitioners did not possess sufficient knowledge about telerehabilitation and had minimal exposure to its implementation. According to Leochico et al., (2022) and Elhadi et al., (2021) rehabilitation experts in their research settings demonstrated insufficient understanding about telerehabilitation with more than 60% of participants showing no knowledge and more than 70% having minimal practical experience.

In contrast, a smaller number of the studies reported encouraging results. Ang-Muñoz et al., (2022) in their study, observed good levels of knowledge among some providers, especially those who had prior exposure to digital health tools. The differences in digital literacy and institutional support and telehealth exposure between regions could be the cause of these inconsistent findings. According to the studies, several factors like age, gender, educational level, experience with electronic devices and applications (Kumar et al., 2022; Zayyad & Toycan, 2018), and geographic location (Sayed & Mamun-Ur-Rashid, 2021) influence knowledge level and play essential roles in determining eHealth knowledge among users.

#### Attitudes Toward Telerehabilitation

The overall attitude of stakeholders towards telerehabilitation appears to be largely positive. Research conducted across various studies shows that healthcare providers and patients want to continue using telerehabilitation services after the pandemic (Abbas et al., 2024; Hossain et al, 2018; Leochico et al., 2021; Pagaling et al., 2022). For instance, Movahedazarhouligh et al. (2015) discovered that Iranian rehabilitation institution faculty members and clinical staff together maintained a 78% favorable view toward telerehabilitation. Technology received exceptional support from occupational therapists and speech therapists. However, these positive attitudes were often tempered by concerns related to privacy and data security. Studies conducted across India, and the Philippines revealed that patients and healthcare providers were apprehensive about the storage and usage of their data (Bajaj & Karuppali, 2022; Koch & Sarma, 2020). According to Xiong et al., (2023) healthcare providers in rural China who showed positive attitudes toward eHealth services required information about data confidentiality, protection and equitable reimbursement to begin service delivery. Service benefits and ease of use were two of the factors that influenced attitudes towards telerehabilitation. According to Alsahli and Hor (2024) physicians’ decisions to use mobile health applications were influenced by social factors and the perceived usefulness and performance expectancy of the application. Similarly, Bhatia (2021) and Hossain et al., (2018) state that telerehabilitation acceptance among people with higher levels of education is higher when they have exposure to smartphones and internet tools.

#### Practices and Adoption of Telerehabilitation

In LMICs the choice of platforms for telerehabilitation often depends on accessibility and ease of use. For instance, a study by Leochico et al (2023) reported that the majority of patients (82.4%) preferred Facebook and a smaller proportion used YouTube, while none opted for WhatsApp, Telegram or Instagram. In contrast, healthcare professionals rely on smartphones, WhatsApp, Zoom, and other videoconferencing platforms for clinical practice (Pagaling et al., 2022). Koch and Sarma (2020) in their study found that videoconferencing is a common practice in physiotherapy, even though some physiotherapists raised concerns that it may not be suitable for conditions such as osteoarthritis. Patient experiences also varied. Kumar et al. (2022) reported that 74% of neurosurgical patients in India preferred audio-consultations over video, citing limited smartphone access and poor 4G connectivity in rural areas. In contrast, Borges et al. (2024) conducted a randomized controlled trial in Brazil involving older adults and found that participants appreciated the accessibility and safety of telerehabilitation, especially as it reduced long waiting times and travel burdens. Moreover, Bhattachariya et al. (2022) conducted one study using an mHealth resource to support the Community based rehabilitation worker (CBR ) workers in implementing adaptive feeding interventions for the children with cerebral palsy. The study found that on-demand digital tools increased CBR workers’ confidence, credibility with caregivers, and the number of families reached, demonstrating that telerehabilitation strategies can enhance adoption and consistent practice of complex interventions in geographically dispersed or resource-limited setting. Adoption and practice are closely tied to user experience and acceptance. Ang-Muñoz et al. (2022), Ong et al. (2022), and Pagaling et al. (2022) adopted the Unified Theory of Acceptance and Use of Technology (UTAUT) model to assess user perceptions and found that knowledge and attitudes are significant predictors of actual use.

#### Reported Recommendations

The themes that have been raised in the recommendations of the selected articles are capacity building for human resources, transparency of funds, policy development, and strengthening the ICT sector (Table 4). The findings emphasized a multi-branched approach to strengthening telerehabilitation services in LMICs. On one hand the capacity building efforts highlighted the need for ongoing training of the service providers, increased awareness and community outreach. On the other hand, the recommendations also stress transparent funding and legal reforms to ensure accountability. Additionally, the call for new policies and guidelines reflects the urgent need to establish clear regulations governing telerehabilitation operations particularly in areas such as data privacy, reimbursement and service standards. Strengthening ICT infrastructure, particularly internet connectivity, security and local language support was viewed as imperative for long term success of this service. Together, these recommendations promote an inclusive, sustainable and context-specific telerehabilitation service.

## Discussion

This scoping review studied the implementation of telerehabilitation together with its challenges and knowledge-attitude-practice levels in LMICs. The review combined data from 84 peer-reviewed articles to show that LMICs transitioned to telerehabilitation mainly because of the COVID-19 pandemic instead of through deliberate planning. The COVID-19 pandemic accelerated the digital rehabilitation service shift by replacing in-person care with virtual sessions, but the reviewed studies indicated that most LMICs failed to provide structural support for this transition. Policies that exist today primarily focus on telehealth without precise guidance for telerehabilitation and disability-specific requirements.

The majority of studies found that most LMICs did not have established national policies or standardized protocols/guidelines or sustainable funding systems for telerehabilitation. The study by Sabrina and Defi (2021) reviewed Southeast Asia and discovered that none of the examined countries had telemedicine-specific laws. Few had clear guidelines about ethical issues and malpractice across borders. This finding closely aligns with the findings of this present review.

The selected literature shows that telerehabilitation implementation barriers in LMICs stem from four primary categories which include technological challenges and organizational barriers together with human factors and legal/ethical considerations. The adoption of telerehabilitation faced multiple technological hurdles that included unstable internet connections, inconsistent power supply, outdated equipment, and insufficient smartphone or telehealth system access. The most severe issues occurred in areas that were both distant from urban centers and lacked sufficient resources because digital inequity remained strong in these regions. Aytur (2024) presented parallel findings in their narrative review about telerehabilitation obstacles for neurological patients. The current review and findings confirm that technological limitations, especially related to internet infrastructure, stand as the primary obstacle for successful implementation.

Organizational barriers, which included change resistance together with resource deficiencies and expensive implementation costs, were documented throughout the literature. According to Sagaro et al. (2020) telecommunication system costs and personnel training expenses and device procurement expenses form major obstacles in LMICs. None of the research included in the current review conducted cost assessments or developed projections for sustaining telerehabilitation programs over time despite financial challenges being a recurring theme. Decision-makers in LMICs depend on cost-efficiency metrics for healthcare funding distribution yet this review discovered that no included studies analyzed costs or developed sustainability models. The absence of economic evaluations presents a major restriction since healthcare funding allocation decisions in LMICs heavily depend on cost-efficiency metrics. The human element plays a crucial role in determining how telerehabilitation services will develop. Multiple research studies indicated that medical staff in LMICs demonstrate insufficient digital competencies and capabilities to use remote service platforms. The professionals maintained a favorable disposition toward telerehabilitation. The 19 knowledge, attitude and practice studies in this review revealed that providers showed willingness to adopt telerehabilitation services if they received appropriate training and institutional backing despite their low initial knowledge. The research by Movahedazarhouligh et al. (2015) along with Bajaj and Karuppali (2022) and Leochico et al., (2021) showed identical findings regarding clinicians’, therapists’, and allied health professionals’ attitudes toward telerehabilitation technology. However, small participant numbers and weak research methodologies limit the general applicability of these findings.

The present review discovered conflicting evidence about factors that affect individuals’ acceptance of telerehabilitation technologies. Bhatia (2021), Kumar et al. (2022), Zayyad and Toycan (2018), and Hossain et al. (2018) found that educational level, gender (Hossain et al., 2018) and age demonstrated significant correlations with technology acceptance. However, Abdulwahab and Zedan (2021) found no such connection. The inconsistent findings between studies indicate that cultural factors together with environmental elements may affect the demographics that influence the rate of technology acceptance and thus demand more adaptive acceptance frameworks.

According to Mohamad and Defi (2022), telerehabilitation challenges in LMICs consist of technical human and administrative barriers. Their research about South and Southeast Asian nations revealed equivalent barriers which stemmed from resistance to change and insufficient ICT skills and inadequate policy support. Furthermore, Erturan et al. (2024) conducted a qualitative survey involving 219 physiotherapists from a developing nation, concluding that telerehabilitation presents an excellent opportunity to enhance healthcare reach. However, they also noted that insufficient digital education and training posed significant barriers to telerehabilitation implementation, similar to the findings of this scoping review.

## Limitations

The research focused on English-language published studies which may result in selection bias. The study may have missed a significant number of publications in languages such as Chinese, South Asian languages, African languages, and South American languages. The study examined LMICs from different continents, yet it did not include enough data from LMICs in Europe, the Middle East and South America.

Articles published before 2012 were excluded from this study which could potentially exclude important information, such as seminal articles from early implementations of telehealth and telerehabilitation. Nonetheless, this review provides essential groundwork for future research while addressing existing knowledge gaps in the field. A systematic evaluation of telehealth services across different LMICs using this review may enable stakeholders and policymakers to create sustainable and useful telerehabilitation programs.

## Conclusion

The scoping review confirms that telerehabilitation in LMICs is an underutilized healthcare delivery approach, but it shows promise. Healthcare providers demonstrate positive attitudes toward telerehabilitation, yet multiple systemic barriers including technological shortcomings, financial constraints, and unclear legal frameworks restrict its complete implementation. The COVID-19 pandemic pushed forward adoption, but numerous countries continue to lack enduring and inclusive strategies for telerehabilitation. The advancement of equitable telerehabilitation requires focused investments to develop technology and train staff while implementing policy reforms. The review demonstrates that low- and middle-income countries need immediate context-based collaborative strategies to provide accessible, affordable, high-quality telerehabilitation services for people with disabilities.

## Figures and Tables

**Table 2:** Country Specific Current Status

Reference	Country	Finance	Purpose	Modality	Media	Available services
Dash et al. (2021)	India	G, P	C, E	R	V, M	Non-COVID service through eShanjeevani
Hoque et al. (2018) Alam et al. (2018) Hossain et al. (2018)	Bangladesh	G, P, F	C, E, S	R	V, M	SMS Electronic health records, Maternity care, 24/7 health helpline service.
Ashfaq et al. (2020) Qureshi et al. (2014) Mahdi et al. (2022) Malik et al. (2024)	Pakistan	G	C, E	R	mHealth based	SMS, mHealth based app, Videoconferencing, Web-based telemedicine
Han et al. (2019)	Sri Lanka	NS	C	R, A	V M	Video conferencing, Audio consultations, Tele-prescriptions Prescriptions sent by SMS
Cruz & Tolentino (2021)	Philippine	G, P	C, S	R, A	M V	SMS, mHealth apps, Use of audio and visual technology for diagnosis
Nguyen et al. (2022)	Vietnam	G	C	A	M	Remote medical service
Afarikumah (2014) Mensah et al. (2023)	Ghana	G F, P	C, E, S	R	M	TeleHealth-related Health- related information via “Healthline”
Sy (2018)	Senegal	G, P	NS	R	M	Hospital Information System, Electronic health record, Telemedicine
Hamad (2019)	Tanzania	G, P	C, E, S	R, A	V, M	Electronic health records, mHealth, Telehealth, Teleradiology,
Shojaee-Mend et al. (2024)	Iran	NS	C, E	NS	NS	Telemedicine Medical information and education provision Diagnosis and treatment/therapy provision

*Note*. G = Government; P = Private/NGO; F = Foreign; C = Clinical; E = Education; S = Surveillance; R = Real Time; A = Asynchronous; V = Videoconference; M = Mobile Phone; NS = Not Specific

**Table 3:** Summary of Barriers and Challenges

Reference	Country	Barriers			
		Technical	Human	Organizational	Legal & Ethical
Hoque et al., (2018) Alam et al., (2018) Choudhury et al., (2024)	Bangladesh	Low electricity supply Less internet access	Users’ acceptability Poor health and technical knowledge Lack of training	Inadequate infrastructure, Financial/Fund issues Resistance to change Lack of policy Lack of promotional activity Corruption Implementation and sustainability cost	Lack of monitoring & accountability.
Chandwani & Dwivediet (2015) Moorthy et al., (2021) Bhatia et al., (2021) Choudhury et al., (2024) Rajkumar et al., (2023) Sengupta et al., (2021) Surya & Someshwar (2025)	India	Low bandwidth Low electricity supply Less internet access Lack of infrastructure	Lack of awareness Lack of practice Shortage of trained personnel Lack of digital literacy Lack of physical examination Poor patient-provider relationship Poor patient participation Affordability and language barrier	Difficulty in booking an appointment Lack of funding Lack of standard assessment quantification Lack of guidelines	Ethical integrity privacy & security Data security Lack of trust
Ashfaq et al., (2020) Qureshi et al., (2014) Mahdi et al., (2022) Choudhury et al., (2024)	Pakistan	Hardware & software issues Less internet access	Users’ acceptability Lack of knowledge Lack of training Shortage of trained personnel Poverty	Inadequate infrastructure Lack of logistic & clinical guidelines Financial/Fund issues Poor eHealth design Cost Lack of tools and technology	Lack of Govt and Stakeholders support Data security. Lack of telehealth laws and regulations
Afarikumah (2014) Mensha et al., (2023)	Ghana	Limitations of access Inadequate ICT infrastructure	Poor health and technical knowledge	Lack of quality measures High Cost	
Kobi et al., (2024)	Cameroon	Lack of reliable electricity Low internet connectivity Lack of infrastructure (rural area)	Lack of training Lack of digital literacy		Lack of guidelines on the integration of telehealth and practice
Hamad (2019)	Tanzania	Lack of compliance	Lack of ICT-related skills & knowledge Lack of training	Inadequate infrastructure lack of governance structure	
Sy (2018)	Senegal		Users’ acceptability Lack of ICT-related skills & knowledge Lack of training Poor Leadership	Corruption Financial/Fund issues	
Dick et al., (2020)	Malawi, Africa	Insufficient ICT infrastructure	Lack of knowledge Lack of affordability of smart phone	Deficiencies in existing health data system	Lack of standards and regulation
Cruz & Tolentino (2021) Reyes et al., (2021)	Philippine	Technical requirement Less internet access	Density of healthcare professionals Resistance to change		Govt legislation and policy Ethical issues
Lamichhane (2020)	Nepal	Insufficient ICT infrastructure	Lack of resources	Sociocultural resources	
Sagaro et al., (2020)	Ethiopia	Insufficient ICT infrastructure Low electricity supply Less internet access Technical support	Lack of ICT related skills & knowledge Lack of awareness Human resource issues	Technically challenged staff, Resistance to change lack of readiness language barrier Poor design and anxiety	
Ezeani et al., (2022)	Nigeria	Insufficient ICT infrastructure Unavailability of the related device High installation cost	Lack of training	No practice regulation	Lack of a legal framework Malpractice
Xiong et al., (2023) Choudhury et al., (2024)	Chaina	Diverse HIT platform	User acceptability Lack of digital literacy	Cost Poor quality of service	Lack of reliability
Tabaeeian et al., (2022) Shojaee-Mend et al., (2024) Choudhury et al., (2024) Ravari et al., (2023)	Iran	Data accuracy problem Lack of support	Lack of training Lack of familiarity with technology Resistance to using technology Lack of awareness and motivation Lack of trust in the therapist for the telehealth practice Difficulties in teaching parents Rehab professionals need to put extra effort in terms of educating parents and vocal strain Limited communication cues	Lack of system integration Indirect cost Lack of funding	Privacy and security concerns Lack of service reliability Lack of autonomy Lack of a definite strategy
Han et al. (2019) Choudhury et al., (2024)	Sri Lanka	Lack of power and electricity at rural area	Poverty	The gap between the private and public sector Indirectness of communication	Privacy and safety
Gregory & Tembo (2017)	Zambia	Information Sharing Complex technology and design	Lack of training	Lack of stakeholders’ support Training laboratories	Lack of adequate policies Lack of Government initiatives
Leochico et al., (2020) Kruse, et al., (2019) Karageorgos et al., (2018) Marzano & Pellegrino (2017) Srivastava et al., (2021) Rabanifar & Abdi (2021) Jones-Esan (2024) Bonnechére,et al., (2023) Ye, He & Beestrum (2023) Dick et al., (2020) Uddin et al., (2024)	LMIC	Insufficient ICT infrastructure Lack of equipment Technology gap Low electricity supply Less internet access Hardware & software issues Lack of Innovation Device & data integration New healthcare system.	Lack of training Users’ knowledge Users’ acceptability Lack of ICT-related skills and knowledge Lack of trained personnel Attitudes of policymakers Shortage of human resources Lack of awareness Lack of affordability	Cost Resistance to change Service level agreement and evaluation criteria Feasibility Language barrier.	Lack of national e- health policies or laws Data privacy Privacy and safety Lack of policy Lack of a standard guideline

**Table 4:** Reported Recommendations

Themes	Key lessons
	
Capacity building for human resources	Ensure technical workshops and training programmes for doctors and consultants (Alam et al., 2021; Dash et al., 2021), nurses, and even patients (Hoque et al., 2018; Kobi et al., 2024)
	Establishment of non-technical training programmes to boost the confidence level of providers (Dash et al., 2021; Hoque, 2016)
	Developing provider and caregiver expertise and experiences with technology (Chandwani & Dwivedi, 2015).
	Awareness development among the users and providers (Hamad, 2019; Qureshi et al., 2014)
	Building capacity of local providers through consultation with remote experts (Dash et al., 2021)
	Design and implement community outreach programmes to enhance awareness (Kobi et al., 2024).
	Conduct outreach services to familiarize people with new technology (Ye et al., 2023)
	Enhance the use of social media to promote public awareness (Ye et al., 2023).
	Encourage multidisciplinary stakeholder collaboration (Dick et al., 2020; Kobi et al., 2024, Surya & Someshwar, 2025)
	
Transparency of funds	Apply funding constraints (Sy, 2018).
	Established, implemented, and followed the “freedom to information” law in LMICs (Ong et al., 2022)
	Evaluation of every implementation phase (Sy, 2018)
	
New policy, protocol, and guideline development	Local telemedicine guidelines need to be established and regulated by higher authorities (Ashfaq et al., 2020)
	Final guideline needs to be prepared asap, which describes the standards and inter-operability procedures of the Health Information Systems Architecture (Alam, 2018)
	Develop Ehealth action lines for an integrated action plan (Sy, 2018).
	Formulation of E-legislation (Hamad, 2019; Oh et al., 2015)
	Develop legal guidelines of equity on accessing patients’ data, maintaining human ethics, and respecting the dignity of man for telemedicine implementation (Rajkumar et al., 2023)
	Integrate digital health in the already established healthcare system (Qureshi et al., 2014, Surya & Someshwar, 2025).
	Formulating rules and regulations to govern telerehabilitation services (Dash et al., 2021)
	Launch a rural people, women, and disability inclusion policy (Sayed & Mamun-Ur-Rashid, 2021)
	Formulation of policy to inform license issues, data privacy, and reimbursement related to teleservices (Kobi et al., 2024).
	Formulation of reimbursement policy to accommodable tele services (Ye, He & Beestrum, 2023).
	Develop interchangeable electronic health records (Ye, He & Beestrum, 2023)
	Develop customized telerehabilitation programs based on socioeconomic, language, and cultural preferences (Surya & Someshwar, 2025)

Strengthening the IT sector	Development of a strong ICT structure (Alejandro et al., 2018; Hoque et al., 2016; Kobi et al., 2024).
	Emphasis on patients’ and providers’ safety and security (Hoque et al., 2018).
	Ensure good bandwidth, excellent network coverage, and network topology that represents the regional needs of a country (Alejandro et al., 2018)
	Provision of infrastructure and technical facilities
	Systems need to be customized to meet the specific demands of patients (Sayed & Mamun-Ur-Rashid, 2021).
	Setting up tools for the natural language process so that the local language can be translated (Qureshi et al., 2014).
	Improve quality and quantity of both tangible and intangible technical factors (Ong et al., 2022; Rajkumar et al., 2023; Ye, He & Beestrum, 2023)

## Data Availability

As this work forms part of a PhD project conducted at an early stage, the data-charting file is not publicly available.

## Appendix

### Glossary of Terms

**Telerehabilitation:** Telerehabilitation has been defined as “the delivery of rehabilitation services via information and communication technologies” and encompasses services that include assessment, prevention, treatment, education, and counselling (Cramer, 2015).

**Low-middle-income countries:** Low-middle-income countries are the nations classified by the World Bank based on the gross national income (GNI) per capita. As of 2024, LMICs are countries with a GNI per capita between 1,146 USD – 4,515 USD (The World Bank, 2025).

### Search Strategies

**Table:**

Terms (keywords & MeSH when available)	

**Concept** (combined with OR)	Telerehabilitation
	Telemedicine
	Telehealth
	ehealth
	mhealth
	(While the primary focus of this study was telerehabilitation, the border term ‘rehabilitation’ was intentionally excluded from the search strategies to enhance precision and minimize the retrieval of non-specific results)

AND	

**Context** (combined with OR)	Developing countries
	Developing nations
	low-middle-income countries
	Third-world countries

AND (removed to enhance the overall search results, as the initial combinations yielded very few relevant papers.	

	People with disabilities
**Population (**combined with OR)	Disabled people
	Service provider
	Service receiver
	Stakeholder

### Database Used

**Table:**

Name of the database (2012–2022)	Initial search result (n) (2012–2022)	Initial search result (n) (2023–2025)
EBSCOhost (CINAHL, Academic Search Premier, MEDLINE)	802	23
PUBMED	496	15
Scopus	318	235
Google Scholar	2590 (full text n=446)	811
Embase	18	0
Cochrane	72	5
Web of Science	1202	506
IEEE Xplore	17	5
LILACS	3	2
AIM	2	0
**Total**	**3376**	**1602**
**Grant total**	**3376+1602= 4978**	

### Summary of Included Articles

**Table:**

Authors (Year)	Country/Setting	Population	Condition/Focus	Modality	Peer-reviewed?	Study Design
Xiong et al. (2023)	China	Patients & doctors	E-health perceptions & utilisation	E-health	Yes	Cross-sectional survey
Tabaeeian et al. (2024)	Iran	Healthcare providers	Barriers to telemedicine use	Telemedicine	Yes	Systematic review
Borges et al. (2024)	Brazil	Older adults on the waiting list for physiotherapy	Telerehabilitation feasibility for discharged older adults	App-based telerehab	Yes	Randomized trail
Rajkumar et al. (2023)	India	Patients/providers in India	Telehealth applications & challenges during COVID-19	Telehealth (mixed modalities)	Yes	Systematic review
Mensah et al. (2023)	Ghana	Health professionals in selected facilities	Readiness for telemedicine implementation	Telemedicine	Yes	Cross-sectional study
Bonnechére et al. (2023)	LMIC	Clinicians, patients, policymakers (review)	mHealth for rehabilitation in LMICs	mHealth (apps, SMS, wearables)	yes	Narrative review
Choudhry (2024)	Pakistan	Regional health systems/stakeholders	Telemedicine prospects & globalization	Telemedicine	Yes	Narrative overview
Alsahli & Hor (2024)	Developing country	Physicians	Adoption of mHealth apps by physicians during COVID-19	Online survey	Yes	Survey/empirical study
Kobi et al. (2024)	Cameroon	Rural healthcare stakeholders	Telemedicine collaboration in rural Cameroon	Telemedicine partnership models	Yes	Case report/project report
Ye, He & Beestrum (2023)	China	Health systems during COVID-19 in China	Implementation & adoption of telehealth	Telehealth	Yes	Systematic review
Malik et al. (2024)	Pakistan	Stroke patients	mHealth applications for stroke rehabilitation	mHealth apps for stroke rehab	Yes	Mini review
Mansouri & Darvishpour (2023)	Iran	Older adults/general populations in COVID context	Mobile Health applications in the COVID-19 pandemic	mHealth (varied)	yes	Scoping review of reviews
Jones-Esan et al. (2024)	LMICs	Not specified	Telemedicine in LMICs	telemedicine	Yes	Systematic review
Shojaee-Mend et al. (2024)	Iran	Health informatics stakeholders	Potential use of digital health in Iran	Digital health (broad mapping)	Yes	Systematic mapping review
Abbas et al. (2024)	Pakistan	Healthcare provider	Acceptance of telemedicine	Telemedicine	Yes	Cross-sectional
Adhikari, Shrestha & Dev (2020)	Nepal	Patients with pain conditions	Pain management via physiotherapy	Telephone-based telephysiotherapy	Yes	Retrospective pre– post design
Afarikumah (2014)	Ghana	Health system stakeholders	e-Health status and prospects	e-Health systems/policies	Yes	Review/descriptive analysis
Alam et al. (2021)	Pakistan	Practicing doctors	Doctors’ perceptions and barriers to telemedicine	Email questionnaire survey	Yes	Quantitative cross- sectional survey
Alam (2018)	Bangladesh	Health stakeholders and systems	mHealth landscape and development	mHealth initiatives	Yes	Review/policy overview
Alboraie (2022)	Egypt	Healthcare providers	Telemedicine adoption and perspectives	Telemedicine services	Yes	National survey
Ang-Muñoz et al. (2022)	Philippines	Medical staff (consultants, residents, fellows)	Telemedicine readiness and acceptance	Telemedicine consultations	Yes	Cross-sectional survey
Ganapathy.(2015)	India	Neurologists and neurosurgeons	Distribution and access to neurological care	Telemedicine adoption	Yes	Cross-sectional survey
Garg et al. (2020b)	India	Public healthcare systems	COVID -19 response and healthcare strengthening	Public health interventions	Yes	Descriptive analysis
Garg et al. (2020a)	India	Healthcare providers and patients	Telemedicine adoption during COVID-19	Telemedicine services	Yes	Cross-sectional survey
Hoque (2016)	Bangladesh	Mobile health service users	mHealth adoption factors	mHealth services	Yes	Empirical study
Dash et al. (2021)	India	Not specified	Telemedicine during COVID-19	Telemedicine services	Yes	Viewpoint
Elhadi et al. (2021)	LMICs	Health worker	Telemedicine awareness, knowledge and skill	Telemedicine services	Yes	Cross-sectional study
Erturan et al. (2023)	Developing country	Physiotherapist	Willingness and challenges of accepting telerehabilitation	Telerehabilitation services	Yes	Web-based survey
Estapé et al. (2022)	LMICs	Mental health cancer care provider	Telehealth and E-health services during COVID-19	Telehealth & E-health services	Yes	Online survey
Kulatunga et al (2020)	Sri Lanka	Not specified	Current state of telehealth	Telehealth practices	Yes	Review article
Hamad (2019)	Tanzania	Not specified	Current state of E-health	Various telehealth services	Yes	Review article
Reyes et al. (2021)	Philippines	Pediatric occupational therapist	Telemedicine in pediatric hospital	Telemedicine services	Yes	Qualitative
Hoque et al. (2018)	Bangladesh	Patients	Adoption of E-health	E-health adoption	yes	Online survey
Mahmoud, Jaramillo, & Barteit (2022)	LMICs	General population	COVID-19	Telemedicine	Yes	Scoping Review
Mahdi et al. (2022)	Pakistan	General population	Various	Telemedicine	Yes	Systematic Review
Movahedazarhouligh & Vameghi (2015)	Iran	Rehabilitation professionals	Rehabilitation	Telerehabilitation	Yes	Cross-Sectional Study
Nguyen et al. (2022)	Vietnam	General population	User acceptance	mHealth	Yes	Qualitative Study
Ong et al. (2022)	Philippines	General population	Telemedicine acceptance	Telemedicine	Yes	Quantitative Study
Oshomoji et al. (2024)	Nigeria	Rural populations	Rehabilitation	Tele-rehabilitation	Yes	Systematic Review
Owolabi et al. (2022)	LMICs	Surgical patients	Surgical conditions	Telemedicine	Yes	Scoping review
Pagaling et al. (2022)	Philippines	Neurology patients	Neurological disorders	Teleneurology	Yes	Cross-Sectional Survey
Qureshi et al. (2014)	Pakistan	Pstients and providers	E-health readiness	e health modalities	Yes	Not specified
Zahid et al. (2017)	Pakistan	Rehabilitation professionals	Rehabilitation	Telerehabilitation	Yes	Commentary
Sagaro et al. (2020)	Ethiopia	Telemedicine implementation	Telemedicine implementation	Telemedicine	Yes	Systematic review
Dick et al. (2020)	Developing countries	mHealth developers/evaluators	mHealth evaluation practices	mHealth (general)	Yes	Retrospective qualitative investigation
Uddin et al. (2024)	LMIC	Tele-neurorehab stakeholders	Enablers of tele-neurorehabilitation	Tele-rehab (general)	Yes	Scoping review
Ravari et al. (2023)	Iran	Speech-language therapists and parents of preschool children	Stuttering telepractice	Synchronous telepractice	Yes	Qualitative (content analysis)
Hossain et al. (2018)	Bangladesh	Rural end-users	eHealth (Portable Health Clinic) adoption	eHealth/telemedicine	Yes	Cross-sectional survey
Sengupta et al. (2021)	India	Parents of autistic children	Online parent-mediated early interventions	Synchronous online intervention	Yes	Pilot feasibility study
Leochico et al. (2020)	Philippines	Adult wheelchair recipients with paraplegia	Telerehab follow-up	Telehealth	Yes	Case report
Tyagi et al. (2019)	India	Individuals with spinal cord injury	Telehealth impact on Quality of life	Telehealth	Yes	Descriptive case report
Surya et al. (2025)	LMICs	Served populations in LMICs	Low-cost telerehabilitation solutions	Telerehabilitation	Yes	Narrative review
Hou et al. (2019)	China	Patients with spinal cord surgery	telerehabilitation for spinal surgery	Mhealth-based rehabilitation	Yes	Randomised control trial
Ezeani et al. (2022)	Nigeria	Patient	Impact of COVID-19	telemedicine	Yes	Prospective feasibility study
Bhattachariya et al. (2022)	India	Community-based rehabilitation worker	Adopted feeding in cerebral palsy	mHealth	yes	Controlled trial
karageorgos et al (2018)	LMICs	Healthcare system	Impact of mHealth	mobile technologies	Yes	Systematic review
Leochico et al. (2023)	Philippines	Community-dwelling persons with stroke	Home-based telerehabilitation	Social media application	yes	Pilot study
Huq et al. (2022)	Bangladesh	Heart failure patients	Telephone-monitored home-based rehabilitation	tele monitoring	Yes	Interventional study
Gregoty &Tembo (2017)	Zambia	Health professionals and systems	E-health implementation challenges	E-health systems	Yes	Case study
Han et al. (2019)	Sri Lanka	Health professionals	m-health adoption perspectives	m-Health technologies	Yes	Qualitative interviews
Chaudhary et al. (2022)	Pakistan & Saudi Arabia	Dental professionals	Teledentistry awareness and challenges	Survey	Yes	Cross-sectional survey
Chandwani & Dwivedi (2015)	India	Healthcare providers	Telemedicine in India	Conceptual analysis	Yes	Conceptual paper
Bajaj & Karuppali (2022)	India	Speech-language pathologists	Telerehabilitation during COVID-19	Telehealth	Yes	Cross-sectional survey
Bhatia et al. (2021)	India	Healthcare users	Telehealth services during COVID-19	Telehealth	Yes	Cross-sectional survey
Biruk et al. (2018)	Ethiopia	Health professionals	Knowledge and attitude toward telemedicine	Telemedicine	Yes	Cross-sectional study
Koch & Sarma (2020)	India	Physiotherapist	Perception of physiotherapists	Telerehabilitation services	Yes	Online-based survey
Kurse et al. (2019)	Developing countries	Patients & providers	Barriers to use mHealth	mHealth services	Yes	Systematic review
Kumar et al. (2022)	Developing countries	Patients who received teleconsultation	Challenges and prospects of teleneurosurgery	teleconsultation	Yes	telephone survey
Lamichhane (2020)	Nepal	Not specified	Impact and challenges of the mHealth application	m health	Thesis	Qualitative study
Macabasag et al. (2016)	Philippines	Not specified	Implementation challenges	telemedicine services	Yes	Review article
Zayad & Toycan (2018)	Nigeria	Healthcare providers and patients	Perspective of health care professionals on e-health adoption	E-health services	Yes	Exploratory survey
Yasmin et al. (2020)	Bangladesh	Type 2 diabetes patients	Diabetes type 2	mHealth	Yes	Randomised Controlled Trial
Sy (2018)	Senegal	Not specified	Current state of Ehealth in Senegal	E-health services	Yes	Not specified
Srivastava et al. (2021)	LMICs	People with neurological disability	Tele neurorehabilitation during COVID-19	Various teleneurological	Yes	Perspective
Sayed & Mamun-Ur- Rashid (2021)	Bangladesh	Patients	Factors Affecting E-Health Implementation	E-health services	yes	Quantitative cross- sectional
Rabanifar & Abdi (2021)	Iran	Studies	Barriers and challenges to the implementation of telerehabilitation	Telerehabilitation services	Yes	Systematic review
Odetunde et al. (2024)	Nigeria	Physiotherapist	Acceptance and adoption of telerehabilitation	Telerehabilitation services	Yes	Mixed Method
Marzano & Pellegrino (2022)	LMICs	Studies	Feasibility & acceptance	Telerehabilitation services	Yes	Scoping review of reviews
Moorthy et al. (2021)	India	Parents and children with disabilities	Telerehabilitation system	Telerehabilitation	yes	Reviewed article
Sharma et al. (2024)	India	Not specified	Barriers and facilitators of telehealth use	telehealth	yes	Systematic review
Leochico et al. (2022)	Philippines	Physiatrists	Perception and experience of telerehabilitation	Telerehabilitation	Yes	online survey
Leochico et al. (2020)	Philippines	Not specified	Challenges in the emergence of telerehabilitation	Telerehabilitation services	Yes	Systematic review
Cruz & Tolentina (2021)	Philippines	Not specified	Telemedicine implementation challenges	Telemedicine	Yes	Literature review
